# The Role of Hyaluronic Acid in Cartilage Boundary Lubrication

**DOI:** 10.3390/cells9071606

**Published:** 2020-07-02

**Authors:** Weifeng Lin, Zhang Liu, Nir Kampf, Jacob Klein

**Affiliations:** 1 Department of Materials and Interfaces, Weizmann Institute of Science, Rehovot 76100, Israel; lin.weifeng@weizmann.ac.il (W.L.); liuzhang@iccas.ac.cn (Z.L.); nir.kampf@weizmann.ac.il (N.K.); 2 Institute of Chemistry, Chinese Academy of Sciences, Beijing 100190, China

**Keywords:** hyaluronic acid, phosphatidylcholine lipids, cartilage lubrication, hydration lubrication, polymer bridging

## Abstract

Hydration lubrication has emerged as a new paradigm for lubrication in aqueous and biological media, accounting especially for the extremely low friction (friction coefficients down to 0.001) of articular cartilage lubrication in joints. Among the ensemble of molecules acting in the joint, phosphatidylcholine (PC) lipids have been proposed as the key molecules forming, in a complex with other molecules including hyaluronic acid (HA), a robust layer on the outer surface of the cartilage. HA, ubiquitous in synovial joints, is not in itself a good boundary lubricant, but binds the PC lipids at the cartilage surface; these, in turn, massively reduce the friction via hydration lubrication at their exposed, highly hydrated phosphocholine headgroups. An important unresolved issue in this scenario is why the free HA molecules in the synovial fluid do not suppress the lubricity by adsorbing simultaneously to the opposing lipid layers, i.e., forming an adhesive, dissipative bridge between them, as they slide past each other during joint articulation. To address this question, we directly examined the friction between two hydrogenated soy PC (HSPC) lipid layers (in the form of liposomes) immersed in HA solution or two palmitoyl–oleoyl PC (POPC) lipid layers across HA–POPC solution using a surface force balance (SFB). The results show, clearly and surprisingly, that HA addition does not affect the outstanding lubrication provided by the PC lipid layers. A possible mechanism indicated by our data that may account for this is that multiple lipid layers form on each cartilage surface, so that the slip plane may move from the midplane between the opposing surfaces, which is bridged by the HA, to an HA-free interface within a multilayer, where hydration lubrication is freely active. Another possibility suggested by our model experiments is that lipids in synovial fluid may complex with HA, thereby inhibiting the HA molecules from adhering to the lipids on the cartilage surfaces.

## 1. Introduction

The articular cartilage coating bone ends in the major joints exhibits extremely low sliding friction (friction coefficient *μ* ≈ 0.001 [1,2,3]) under physiologically high pressures (up to 1 × 10^7^ Pa or more [4,5]). Both cartilage fluid pressurization [1,6,7] and boundary lubrication [3,8,9,10,11] are responsible for this superior lubricity. Over the past two decades, the concept of hydration lubrication has been an emergent paradigm in understanding the low friction that may be observed at high pressures between charged surfaces in aqueous media [12,13,14,15,16]. This arises from tenaciously bound hydration layers around charges, which can support large pressures without being squeezed out, while remaining highly fluid: this combination of maintaining high loads while sliding with little frictional dissipation results in very low friction coefficients. A particularly interesting finding within the hydration lubrication paradigm is that highly hydrated phosphocholine groups, the headgroups of phosphatidylcholine (PC) lipids, the most common in our body and abundant in cartilage and synovial fluids [17,18], may act as exceptionally good boundary lubrication elements. PC layers at sliding interfaces exhibit extremely efficient lubrication properties (friction coefficient *μ* down to 10^−4^ or less) at pressures up to over 1 × 10^7^ Pa [19,20,21]. It was found that lipids in their gel phase (such as hydrogenated soy PC (HSPC), whose main phase transition temperature *T*_m_ = 53 °C) were far more robust lubricants than liquid state lipids (e.g., palmitoyl–oleoyl PC (POPC), *T*_m_ = −20 °C) in lipid-free aqueous media due to the lower structural integrity of POPC lipid layers. In contrast, POPC lipid layers provide excellent lubrication up to high pressures when POPC is present in the surrounding aqueous medium, due to the rapid healing of its more fluid bilayers in the presence of free lipids [20,22,23].

Hyaluronic acid (HA), a negatively charged, nonsulfated glycosaminoglycan ubiquitous both in synovial fluid and articular cartilage [24,25], was for many years, assumed to be the molecule responsible for cartilage lubrication [26,27]. Clearly, HA plays a major role in joint homeostasis [28,29,30]; however, its boundary lubricating ability is poor, as directly determined experimentally by surface force balance (SFB) investigations [31,32,33]. Recent studies by Seror et al. [33] and Zhu et al. [34] demonstrated that the friction between two surface-attached HA layers was much reduced (from *μ* ≈ 0.3 to *μ* ≈ 0.001) after adding small unilamellar vesicles (SUVs) composed of PC lipids.

**Figure 1:**
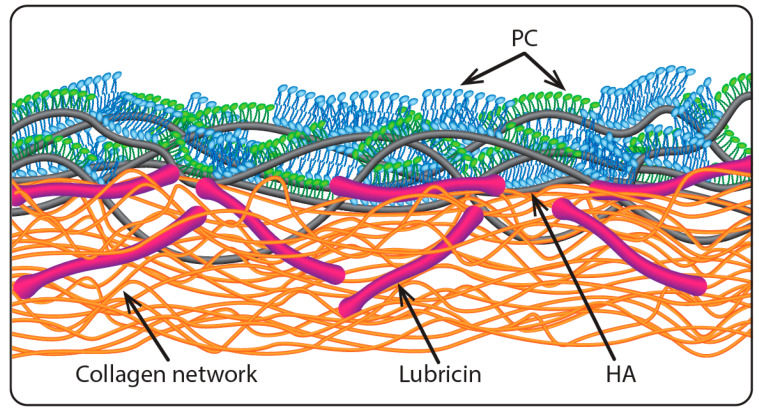
Illustrating the proposed boundary layer on articular cartilage [33], where lubricin molecules anchor hyaluronic acid (HA) chains at the articular cartilage surface and the HA is complexed with phosphatidylcholine (PC) layers exposing their highly hydrated phosphocholine groups at the very outer surface (the slip plane). Reproduced with permission from [33]. Copyright 2016, Annual Review.

In the context of biolubrication, PC lipids are well known to interact with HA molecules [35,36,37,38,39,40,41,42,43]. A mechanism for boundary lubrication of articular cartilage was proposed by Seror et al. [33] based on their study showing that gel-phase PC lipids complexed with surface-attached HA molecules to provide strong boundary lubrication. According to this, PC lipids, HA, and lubricin act together, as illustrated in Figure 1: lubricin attaches HA at the cartilage surface, and PC lipids complex with the HA molecules, exposing their highly hydrated phosphocholine groups at the slip plane between opposing cartilage surfaces, to provide the extremely low friction [3,33] via the hydration lubrication mechanism. While lipid bilayers and monolayers are shown in the cartoon in Figure 1, multilayers of the lipids or indeed intact liposomes may be attached to the HA molecules, as seen directly in other studies [34,36].

The proposed cartilage boundary layer illustrated in Figure 1 poses an immediate conceptual problem. Given that HA is ubiquitous and at relatively high concentrations in the synovial fluid which fills the joint cavity, one would expect HA molecules to attach to the exposed lipid layers from the synovial fluid [44,45,46], and more importantly to attach simultaneously to the exposed lipids on both opposing cartilage surface, bridging the gap between them. Such polymer bridges are known to greatly increase the sliding friction between surfaces, as during sliding the chains are dragged across the opposing surfaces, leading to considerable energy dissipation as bonds are broken and reformed [47,48]. The crucial question then arises whether, in view of such bridging by HA, the proposed boundary layer (as illustrated in Figure 1) is indeed capable of providing the low friction associated with hydration lubrication between the exposed, hydrated phosphocholine groups at the slip plane. In order to answer this question, we examine here directly the lubrication between two lipid layers (saturated and unsaturated PC lipids, in their gel and liquid phases respectively) immersed in HA solution or in an HA/lipid mixture, compared to immersion in lipid-free water or in lipid dispersion, using an SFB. Our experiments reveal that the efficient lubrication is indeed retained between surface layers of PC lipids, even in the presence of HA molecules. Two possible mechanisms are suggested to explain this unexpected result.

## 2. Materials and Methods

### 2.1. Materials

Hydrogenated soy phosphatidylcholine (HSPC; 16:0, 15%/18:0, 85%, PC) and 1-palmitoyl-2-oleoyl phosphatidylcholine (POPC; 16:0/18:1, PC) were purchased from Lipoid (Ludwigshafen, Germany). Hyaluronic acid (HA) with a molecular weight of 1.5 × 10^6^ g/mol (with a polydispersity of 2.1) was purchased from Creative PEGworks (Durham, NC, USA). Materials were used as received. The water used throughout the experiments was purified by an activated charcoal filter and a Barnstead Nanopure system, with a total organic carbon content of ca. 1 ppb and a resistivity of 18.2 MΩ·cm. Mica (ruby muscovite, grade 1) was purchased from S&J Trading (New York, NY, USA). EPON^TM^ epoxy resin 1004 used to glue the mica sheet onto the glass lens in the SFB experiments was purchased from Miller-Stephenson Chemical (Danbury, CT, USA).

### 2.2. Preparation of Liposome and Hyaluronic Acid Solutions

Small unilamellar vesicles (SUVs) were prepared as described previously [42,49]. Briefly, lipids were ultrasonicated in water for 15 min at a temperature above the phase transition temperature (*T*_m_) of the lipid (*T*_m_ (HSPC) = 53 °C; *T*_m_ (POPC) = −2 °C) and vortexed for 1 min in order to yield multilamellar vesicles (MLVs). The obtained MLVs were gradually downsized to SUVs using a Lipex^TM^ extruder (Northern lipid Inc., Burnaby, BC, Canada) through hydrophilic polycarbonate membranes (Avanti Polar Lipids, Inc., Alabaster, AL, USA), with pore sizes from 400 nm (5 cycles), to 100 nm (8 cycles), and ending at 50 nm (12 cycles). Hyaluronic acid was dissolved in pure water (pH ~6.0) at a concentration of 0.5 mg/mL. The HA–POPC mixture was prepared by mixing HA (1 mg/mL) and POPC SUVs (0.6 mM) (1:1, *v*/*v*) under stirring at room temperature for 48 h.

### 2.3. Dynamic Light Scattering (DLS)

**Figure 2:**
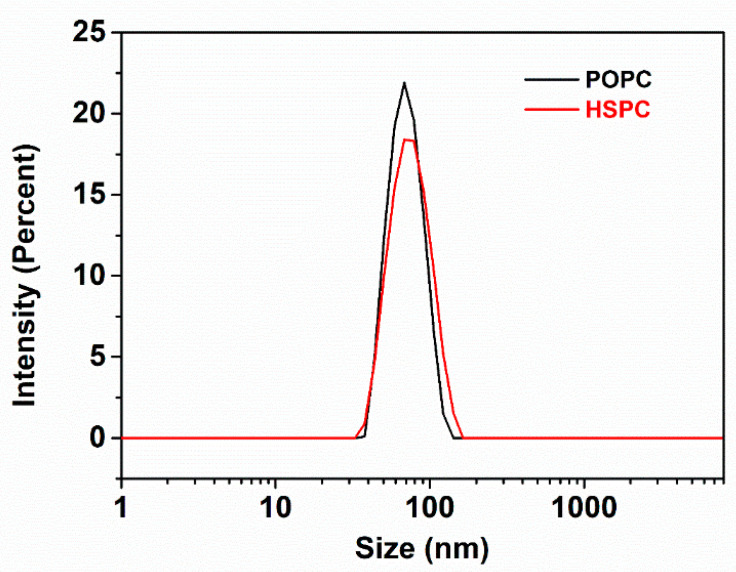
Size distribution of HSPC and POPC SUVs in water determined by dynamic light scattering (DLS). The hydrodynamic size and polydispersity index (PDI) for HSPC and POPC SUVs in water are 73 ± 6 nm (PDI = 0.071 ± 0.015) and 68 ± 5 nm (PDI = 0.056 ± 0.021), respectively.

The size distributions of the SUVs were characterized in pure water (1 mM) by a Zetasizer Nano instrument (Malvern Instruments, Malvern, United Kingdom) at a scattering angle of 173° and a temperature of 25.0 ± 0.1 °C. The dynamic light scattering (DLS) results (three measurements for each sample) indicated that the hydrodynamic sizes of HSPC and POPC liposomes in water were 73 ± 6 nm and 68 ± 5 nm, respectively (as shown in Figure 2). The size (*D*_h_) was determined from diffusion coefficients (*D*) by the Stokes−Einstein relation for a sphere, *D*_h_ = *k*_B_*T*/3π*ηD*, where *k*_B_ is the Boltzmann constant, *T* is the absolute temperature, and *η* is the viscosity of the solvent. 

### 2.4. Atomic Force Microscope (AFM) Imaging

**Figure 3:**
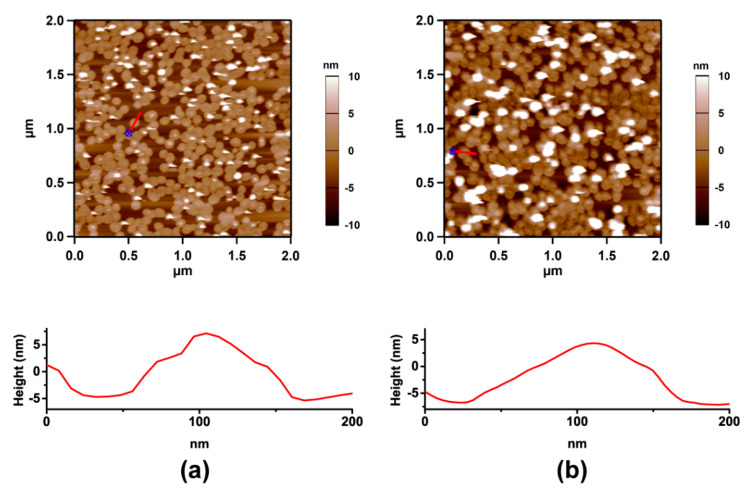
Atomic force microscope (AFM) images of HSPC SUVs adsorbed on mica under water (**a**) and HA solution (**b**). The height distance cross section of the surface along the red line is presented below each image.

Atomic force microscope (AFM) imaging was carried out using an Asylum MFP-3D standalone AFM (Oxford Instruments, Santa Barbara, CA, USA). The sharp nitride lever (SNL) tip (SNL-10, a silicon tip with a 0.35 N/m spring constant on a silicon nitride cantilever, Bruker AFM Probes, Camarillo, CA, USA) was used with tapping mode in water or the HA solution at room temperature (as shown in Figure 3).

### 2.5. Surface Force Balance (SFB) Measurements

**Figure 4:**
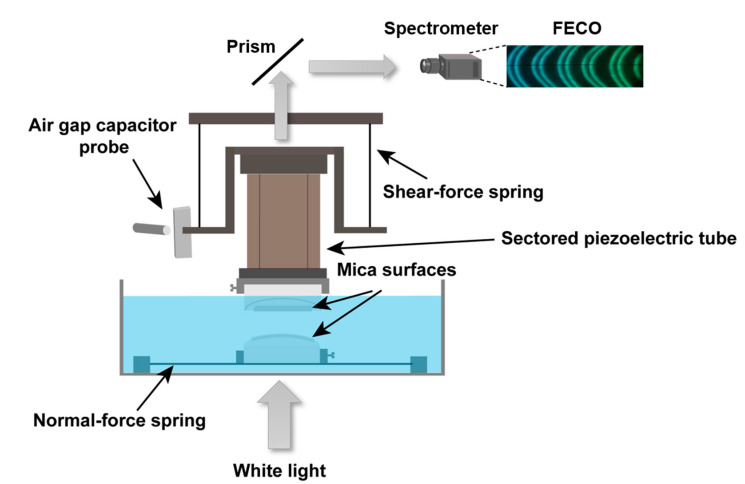
Schematic illustration of the surface force balance (SFB) setup, where normal and shear forces between two surfaces are directly measured via the bending of two orthogonal springs. White light undergoes multiple beam interference in passing through the half-silvered mica sheets, resulting in Fringes of Equal Chromatic Order (FECO, top right inset). These yield absolute separation between the surfaces to within ±0.2 nm. A sectored piezoelectric tube can move the surfaces normally and laterally relative to each other. The bending of normal and shear springs in response to applied motion, monitored via the change in an air gap capacitor or through changes in the interface fringes, directly reveals the shear and normal forces, respectively.

The SFB technique has been described in detail previously [50,51,52], and the setup is shown schematically in Figure 4. Briefly, the molecularly smooth mica sheets were prepared by the melt-cutting method [53]. Mica sheets half-silvered on their backsides (thickness ~2–4 μm and size ~1 cm × 1 cm) were glued on plano-cylindrical fused silica lenses. The mica-coated lenses were amounted in the SFB in a crossed-cylinder configuration. The separation *D* between the mica surfaces and the mean radius of curvature *R* (≈10 mm) were measured by monitoring the optical interference fringes of equal chromatic order (FECO) when a beam of white light was transmitted through back-silvered mica sheets. The normal force *F*_n_ with a sensitivity of 10 nN is calculated as *F*_n_ = *K*_n_∆*D*, where *K*_n_ is the normal force spring constant (127 N/m) and ∆*D* is the bending of the normal spring; while the shear force *F*_s_ *= K*_s_∆*x* is determined by monitoring the bending of the two orthogonal springs (∆*x*) using an air gap capacitor, where the shear force spring constant *K*_s_ = 304 N/m. The bare mica surfaces after calibration were incubated overnight within the SFB boat in a liposomal dispersion (0.3 mM, for both HSPC and POPC). In the case of HSPC, surface forces were measured under POPC dispersion, then the dispersion was removed (the surfaces didn’t pass through the air-water interface since the meniscus was kept), and replaced by a HA–POPC mixture (0.5 mg/mL: 0.3 mM) and forces measured. At each surface separation, the lateral back-and-forth motion of the top (piezo-mounted) surface was carried out for 1 min, and the traces directly monitoring the friction forces transmitted to the lower surface were recorded. The results shown are based on 5 different independent experiments and different contact points within each experiment. Note that for normal force profiles *F*_n_(*D*), which are the normal forces acting between the two curved mica surfaces for the closest separation *D* apart, the results are normalized as *F*_n_*(D)*/*R*, where *R* is the mean radius of the curvature of the mica surfaces. In the Derjaguin approximation (valid in this case since *D* << *R*), this yields the interaction energy per unit area of interaction for flat parallel surfaces obeying the same force law.

## 3. Results

In this study, layers of PC lipids were created on model substrates (mica), and the normal and shear forces between them were examined directly in the SFB, both in the absence and in the presence of polysaccharide HA in the surrounding solution. The results shown are based on five independent experiments, with at least two independent experiments for each lipid (including several independent contact points within each experiment).

### 3.1. Normal Forces

**Figure 5:**
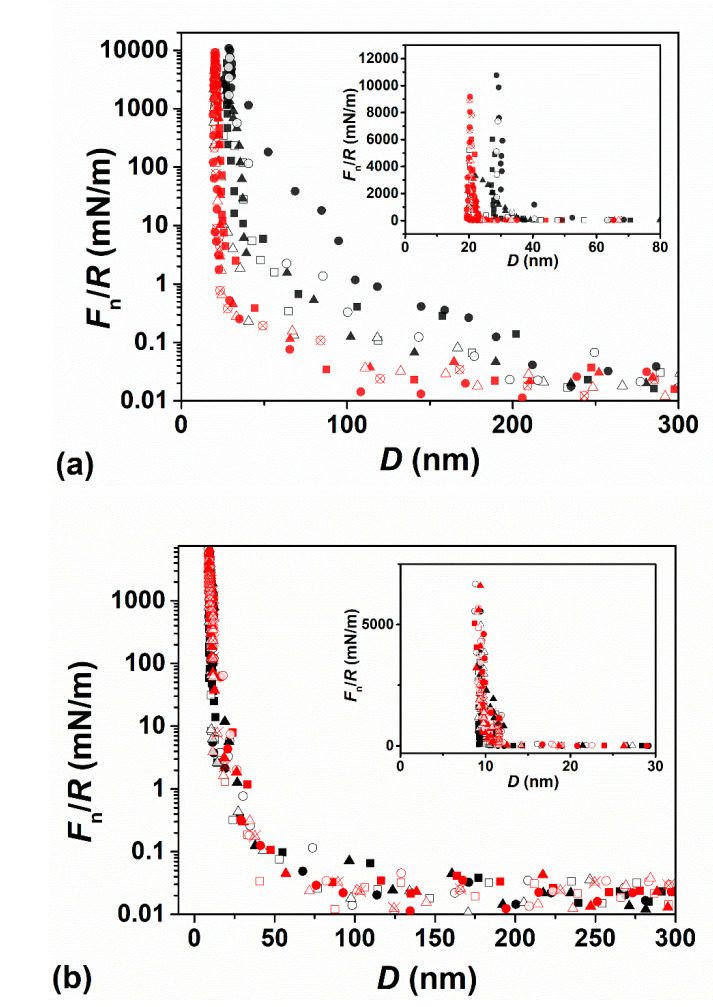
(**a**) Normal force profiles *F*_n_(*D*)/*R* versus separation *D* between two mica surfaces across pure water (black symbols) following overnight incubation in HSPC small unilamellar vesicle (SUV) dispersion (0.3 mM) followed by washing, and thereafter across HA solution (red symbols), normalized in the Derjaguin approximation by the mean radius of curvature of the mica sheets (*R*). (**b**) Normalized force profiles *F*_n_(*D*)/*R* versus separation *D* between two mica surfaces across POPC dispersion (0.3 mM, black symbols) following overnight incubation, and then across the HA–POPC mixture (0.5 mg/mL:0.3 mM, red symbols). The zero of separation (*D* = 0) is with respect to air contact between bare mica surfaces. The insets show the ‘hard wall’ behavior on a larger, linear–linear scale with the same data points as in the main figure; in (**a**) the hard wall in HA-free water (black symbols) is generally at ca. 30 nm (and occasionally at ca. 20 nm), and in HA solution it is consistently at ca. 20 nm; in (**b**) the hard wall is at ca. 10 nm in both POPC dispersion and in the HA–POPC mixture. Different shaped symbols correspond to different contact positions based on 5 independent experiments with first approaches (filled symbols), second approaches (empty symbols), and receding profiles (crossed symbols).

Prior to adsorption of the liposomes, interactions between the two opposing bare mica surfaces in air was measured, to ensure that the mica is not contaminated, and to determine the mica-mica zero separation. The two bare mica surfaces were then incubated overnight in HSPC SUV dispersion (0.3 mM) within the SFB bath, and following which the dispersion was replaced by pure water. As the two surfaces approached from ca. 500 nm, no interaction was detected above the scatter at surface separations *D* > ca. 200 nm. Force profiles of HSPC SUVs coated mica surfaces in pure water, Figure 5a, indicated that *F*_n_(*D*) commenced around ca. 150–200 nm. Considering that the zwitterionic lipid headgroups are neutral, this indicates that steric repulsion dominates between opposing liposome layers at this separation due to an over-layer of loosely attached HSPC SUVs on top of a surface-attached lipid layer, in line with similar observations in the previous studies [20,22,54]. Upon strong compression, ”hard wall“ separations of 20 ± 3 nm and (mostly) 28 ± 3 nm were obtained (see inset to Figure 5a), corresponding respectively to 2 and 3 compressed liposomes layers (the thickness of one bilayer and one flattened SUV layer is ca. 5 nm and ca. 10 nm [22,55]). The force profiles of HSPC SUV-coated mica surfaces in HA solution (Figure 5a) showed that the repulsion set off from ca. 100 nm, which was significantly closer than in pure water. This may be attributed to the HA molecules in the bulk removing some of the loosely bound liposomes; the ‘hard wall’ separation also decreased to 20 ± 3 nm (see inset of Figure 5a), likewise indicating the presence of fewer liposomes in any over-layer. Moreover, as the surfaces separate, there is no indication of adhesion between them; such adhesion might arise if HA molecules were bridging the gap between the surfaces (adsorbing simultaneously on both opposing lipid surfaces). Such an absence of adhesion may be due to steric repulsion from the compressed, confined HA molecules which is much higher than any weak dipole-charge bridging interaction.

In the second lipid system studied, the bare mica surfaces were incubated overnight in a POPC SUV (0.3 mM) dispersion and the surface forces measured. This dispersion was then replaced by a POPC-HA mixture, incubated overnight again, and the surface forces measured again. Force profiles are shown in Figure 5b. Normalized force profiles *F*_n_(*D*)/*R* versus separation *D* across POPC SUV dispersion (0.3 mM) and across the HA–POPC mixture are shown in Figure 5b. Since POPC bilayers readily rupture when compressed and sheared under pure water [20,23], force profiles were measured under POPC SUV dispersion (with and without added HA), where the layers are stable under compression and shear due to the rapid healing of the fluid bilayers in the presence of free lipids [23]. Weak repulsion sets on at separations in the range ca. 75–100 nm, attributed largely to loosely attached liposomes on the surface-attached POPC bilayers, but these are removed on approach more easily than HSPC SUVs. A “hard wall” is observed at a separation of 9.1 ± 1.0 nm (see inset of Figure 5b), which corresponds to the thickness of two lipid bilayers. There was no significant difference between force profiles measured across the POPC dispersion and across HA–POPC mixture (0.5 mg/mL: 0.3 mM, Figure 5b—red symbols) once HA was introduced. We may conclude the HA–POPC complex [34,56] was not trapped between the surface, but rather that it was squeezed out between POPC bilayers as the surfaces approached.

### 3.2. Shear (Frictional) Forces

**Figure 6:**
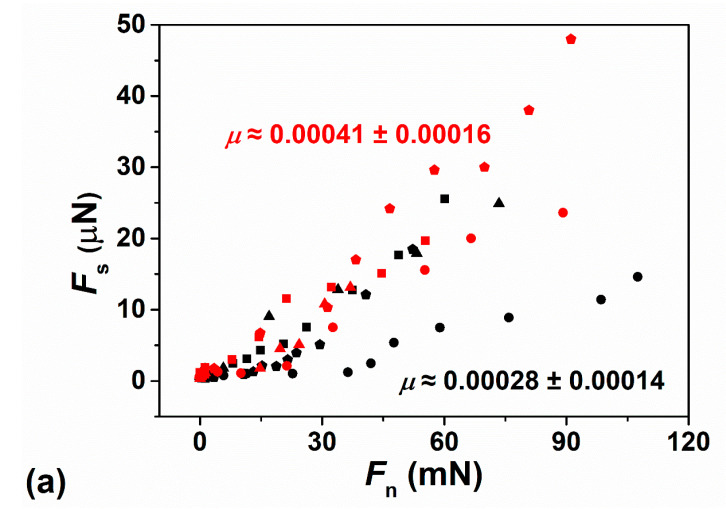

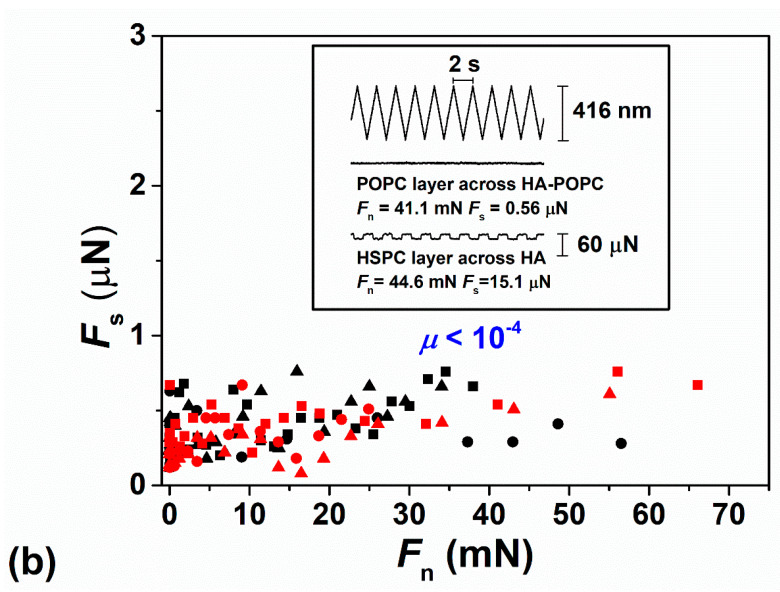
(**a**) Frictional force *F*_s_ versus *F*_n_ between two mica surfaces across pure water following overnight incubation with HSPC SUVs dispersion (black symbols), where *μ* ≈ 0.00028 ± 0.00014, and across HA solution (red symbols), where *μ* ≈ 0.00041 ± 0.00016. (**b**) Frictional force *F*_s_ versus *F*_n_ between two mica surfaces across POPC SUVs dispersion following overnight incubation with POPC SUVs dispersion (black symbols) and across POPC-HA mixture (red symbols), where both of them showed *μ* < 0.0001. The inset shows directly measured shear traces *F*_s_(*t*) for POPC layer across HA–POPC (2nd trace) and HSPC layer across HA (3rd trace) with similar normal force as a function of applied sliding motion (top trace). The low values of *F*_s_ are determined by fast Fourier transform of the shear traces [12].

The shear (frictional) forces *F*_s_ transmitted between the interacting surfaces are measured by monitoring the bending of the shear springs (Figure 4) as the upper surface moves laterally with respect to the lower surface. Typical applied lateral back-and-forth motions (top trace) and corresponding shear traces—from which *F*_s_ is extracted—at different surface separations and normal loads are shown in the inset image of Figure 6b. The variations in shear force *F*_s_ with *F*_n_ between two mica surfaces following overnight incubation with HSPC SUV dispersion, across pure water and across HA solution, are presented in Figure 6a, where the friction coefficient *μ = F*_s_/*F*_n_ ≈ 0.00028 ± 0.00014 for HSPC layer in pure water, and *μ* ≈ 0.00041 ± 0.00016 for HSPC layer in HA solution. In the case of POPC, *μ* < 0.0001 was found with the applied force in the study for two mica surfaces across POPC SUVs dispersion following overnight incubation with POPC SUVs dispersion and across HA–POPC mixture. These exceptionally low friction coefficients between two mica-supported vesicle layers or bilayers are in line with values that have been previously observed between mica sheets bearing HSPC-SUVs across water [20], and bearing POPC-SUVs across POPC-SUV dispersion [23]. The low friction in those earlier studies was attributed to the hydration lubrication mechanism acting between the exposed, highly hydrated phosphocholine headgroups of the PC lipids. The present results show similarly low friction, but, crucially, little difference whether or not HA is present: this is a puzzling observation, since HA is known to interact with PC lipids so that it might be expected to bridge the gap between opposing PC layers and thus increase sliding friction; we consider the reasons for this in the following section.

## 4. Discussion

Model substrates—such as molecularly smooth mica—are clearly different to articular cartilage, but they allow close control over surface roughness and the detailed nature of the boundary layers. For this reason, they may provide insight at the molecular level into the mechanisms of friction which occur at the slip plane (boundary lubrication), and which are largely independent of the substrate. Cartilage, once removed from the joints, no longer resembles in-vivo cartilage; so it is difficult to compare the lubrication of such explants with what happens in real joints. At the same time, while cartilage explants more clearly resemble cartilage in their bulk properties [57], it is difficult to control their precise surface boundary composition and roughness. Friction arises from the energy dissipation at the slip plane between the two surface boundary layers as they slide past each other, which in turn is due to the different molecular interactions, including dipole-charge interaction, van der Waals and hydrophobic interactions, and rupture and reforming of hydrogen bonds. In the hydration lubrication paradigm surfaces interact via fluid but tenaciously attached hydration layers exposed by their boundary layers at the slip plane, resulting in low friction as surfaces slide past each other [12,58]. Previous studies [34,35,36,37,38,39,40,41,42,43,56,59,60,61,62,63,64] showed that HA interacts with PC lipids when they are dispersed in aqueous solution or when the phospholipids are incubated with pre-adsorbed HA on a surface, due to charge-dipole interactions between the negatively charged HA and the exposed zwitterionic phosphocholine headgroups. Thus one would expect that HA would adsorb at a phospholipid-bilayer/water interface. In such a case, HA molecules would attach on both opposing PC layers as the mica surfaces (on which they are adsorbed) approach, to form polymer bridges between them. When the surfaces slide past each other, the HA bridges drag across the surfaces, dissipating energy and resulting in higher friction, as has been observed in other cases of polymer bridging [47,48,65]. However, the central, and surprising, finding of the current study, where both saturated (HSPC) lipids in their gel (solid-ordered) phase and unsaturated (POPC) lipids in their liquid phase are investigated, indicates that this is not the case: the friction remains equally low with or without HA. Why is this?

**Figure 7:**
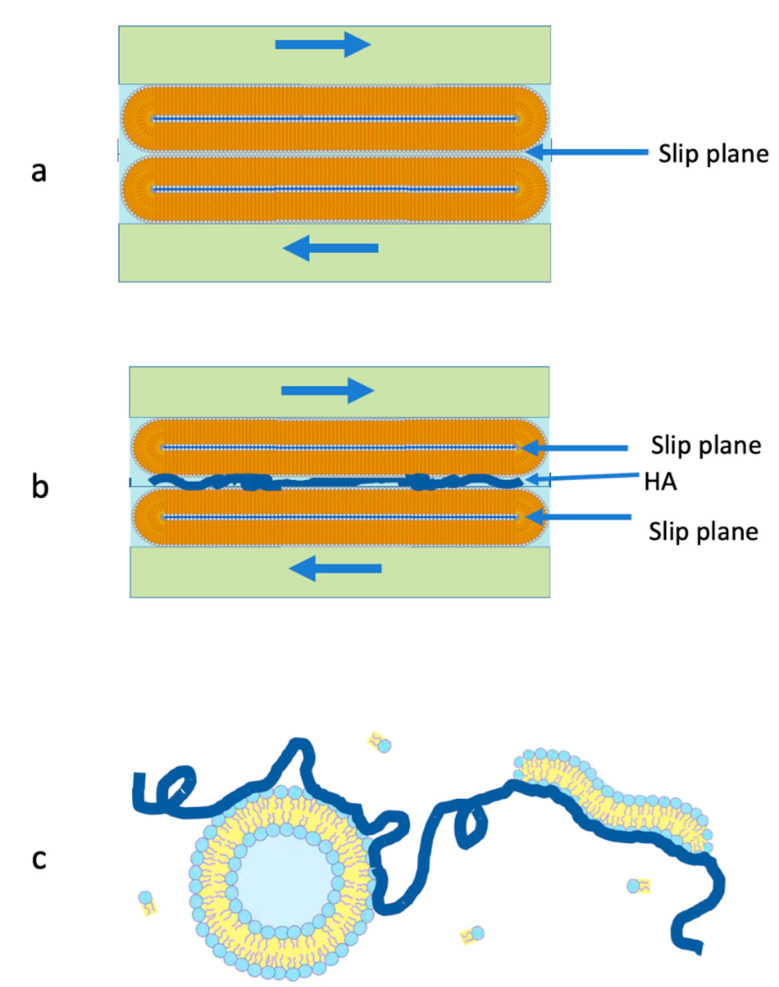
Illustrating the two mechanisms which are attributed to enable low friction hydration lubrication between PC-coated layers, even in the presence of HA. (**a**) In the case of the gel-phase lipid layers (HSPC) in HA-free water, the slip plane is the mid-plane between the vesicles. (**b**) When HA is added, it may adsorb on the vesicles’ surfaces to bridge the gap between them, leading to high friction at the mid-plane interface. As a result, the slip plane shifts, as shown, to the inner interfaces, where hydration lubrication is unhindered by the HA bridging. (**c**) In the case of the liquid-phase POPC, where the surfaces are immersed in a POPC dispersion (see text), the addition of HA leads to interactions between the lipids in solution and the polysaccharide, as indicated; this suppresses their adsorption on the surface-attached POPC bilayers and thus enables hydration lubrication between them, unhindered by any HA bridging.

It is known that saturated lipid layers (like HSPC) organize as close packed bilayer vesicles on mica surfaces [20]. During shearing, two such layers are compressed and slide past each other, the slip plane between them is between the outer leaflets on either surface, as illustrated schematically in Figure 7a. In the presence of HA, we expect the polysaccharide to adsorb and bridge the outer leaflets, as illustrated in Figure 7b. In that case, friction across the bridged mid-plane interface will be higher, as discussed above, and we attribute the low observed friction to the shift of the slip plane to either of the two internal interfaces indicated in Figure 7b: the highly hydrated phosphocholine headgroups of the inner liposome leaflets spanning the new slip planes will be hydration lubricated as they slide past each other. There is, indeed, some indication (Figure 6a (red data points)) of a small increase in *μ*, on average, when HA is present, relative to the HA-free case (black data points), though the friction remains low and well within the so-called superlubricity regime (*μ* < 0.01) [55].

The case of the unsaturated POPC is different. Vesicles of this lipid in its liquid-disordered phase rupture to form flat bilayers when adsorbed on mica. High friction is measured already at low contact stresses when shear is applied between two such POPC bilayers immersed in lipid-free water [20], an effect attributed to their degradation under shear occurs due to the lower robustness of the liquid-phase layers relative to bilayers of lipids (such as HSPC) in their gel phase. When POPC bilayers are immersed in the dispersion of the lipid, however (in the form of POPC-SUVs), it is found [23] that the surface-supported bilayers act as excellent lubricating boundary layers up to physiologically high pressures (of order 1 × 10^7^ Pa), attributed to the rapid healing of the layers by the available surrounding lipids. This is why measurements with POPC in this study are carried out in a POPC dispersion. Nonetheless, the presence of any bridging HA molecule would be expected to result in higher friction, due to dissipation arising from drag of the polymer across the sliding surfaces. The fact that addition of HA to the surrounding POPC dispersion does not result in such increased friction, as seen in Figure 7b, cannot be due to the shift of the slip plane (as with HSPC above), since there is only a single interface, at the mid-plane, where hydration lubrication between phosphocholine headgroups can be active. On the other hand, it is attributed to the interaction of the HA molecules in the HA/POPC mixture with free POPC lipids in the mixture, or possibly with the vesicles themselves, as illustrated in Figure 7c. Once the HA attaches to POPC in the surrounding bulk as indicated schematically in Figure 7c, strongly reducing its available lipid-attachment sites, its tendency to adsorb onto the POPC bilayers attached to the surface is much reduced. Thus, as the surfaces approach, the ”lipid-encrusted“ HA molecules will be expelled from the gap for entropic reasons; therefore, they will not adsorb to form bridges and hydration lubrication will occur unhindered between the POPC bilayers, providing the low friction, as indeed observed (Figure 6b).

## 5. Summary and Conclusions

We examined the effect on the lubrication by surface-attached PC lipids, in the form of either vesicles or bilayers, of introducing HA, which is known to interact via charge–dipole forces with such lipids, and so might be expected to form friction-enhancing bridges between the lipid-coated surfaces. Surprisingly, on adding HA there was little change in the friction, which remained persistently low as a result of hydration lubrication between the highly hydrated phosphocholine headgroups exposed by the lipid layers. This was attributed to different effects: for the case of the HSPC vesicles on the surfaces, bridging may occur but the resulting increased friction at the mid-plane may be overcome by the shift of the slip plane to internal lipid–lipid interfaces, which are free of any HA-bridging effects, and thus are well hydration lubricated. For the case of liquid-phase POPC bilayers, which lubricate well only in a POPC dispersion (which enables the healing of the bilayers if they are shear degraded), we attribute the persistent good lubrication to interactions of the added HA with lipids in the surrounding bulk dispersion. Such bulk-lipid/HA interactions suppress its adsorption on the surface-attached POPC bilayers, and enables unhindered hydration lubrication between them. While our experiments were carried out on model systems, where the substrates are atomically smooth, rigid mica surfaces, we believe nonetheless that our conclusions also have marked relevance for synovial joints, where articular cartilage is lubricated by PC lipid boundary layers, while the surrounding synovial fluid contains both lipids and HA. This is because the boundary layers on cartilage, which are neither smooth nor rigid, are likely to expose PC multilayers rather than a single bilayer [3,34,66], so that if bridging by HA occurs at the outer PC layers, the slip plane may readily shift to internal interfaces within such multilayers, as illustrated in Figure 7b. Likewise, HA, which is abundant in synovial fluid, may interact with PC lipids, which are also ubiquitous in the fluid, thereby suppressing its tendency to attach to the PCs exposed at the cartilage boundary layers. In conclusion, our results show, unambiguously, that the presence of HA does not hinder hydration lubrication between surface-attached PC layers.
